# Comparison between frequency-doubling technology perimetry and standard automated perimetry in early glaucoma

**DOI:** 10.1038/s41598-022-13781-2

**Published:** 2022-06-17

**Authors:** Seong Ah Kim, Chan Kee Park, Hae-Young Lopilly Park

**Affiliations:** Department of Ophthalmology and Visual Science, Seoul St. Mary’s Hospital, College of Medicine, Catholic University of Korea, 505 Banpo-dong, Seocho-ku, Seoul, 137-701 South Korea

**Keywords:** Ocular hypertension, Optic nerve diseases

## Abstract

This study aimed to find out the significance of the difference between frequency-doubling technology perimetry (FDT) and standard automated perimetry (SAP) in terms of the detected visual field (VF) damage, and evaluate associated factors to SAP–FDT difference in early glaucoma. Glaucoma patients in early stage (MD better than − 6.0 decibel, 96 eyes) were included in this cross-sectional study. We subtracted mean deviation (MD) and pattern standard deviation (PSD) of FDT from those of SAP, respectively. Additionally, we counted significantly depressed points of *P* < 5% and *P* < 1% on the pattern deviation probability plot of both FDT and SAP and defined eyes with significant SAP–FDT difference when the number of abnormal points were greater than 4 points on FDT. We measured lamina cribrosa depth (LCD) and lamina cribrosa curvature index (LCCI) for structural parameters of the optic nerve head from images using enhanced depth imaging of the optical coherence tomography (OCT). Peripapillary vessel density (VD) and presence of microvasculature dropout (MvD), the complete loss of choriocapillaris in localized regions of parapapillary atrophy, was evaluated using deep layer map of OCT angiography (OCT-A) for vascular parameters. Peripheral nasal step (PNS) group had an isolated glaucomatous VF defect within nasal periphery outside 10° of fixation. Parafoveal scotoma (PFS) group had an isolated glaucomatous VF defect within 12 points of a central 10˚ radius. Eyes with significant SAP–FDT difference showed higher detection of MvD on deep layer map of OCT-A, greater LCD, and greater LCCI (all *P* < 0.05, respectively). In logistic regression analysis, frequent presence of MvD, less presence of disc hemorrhage, and greater LCD were significantly associated with significant SAP–FDT difference. Sub-analysis was performed in eyes with PNS (50 eyes) and PFS (46 eyes). SAP–FDT difference of MD value showed positive association with peripapillary VD on deep layer of OCT-A, which was significant in eyes with PFS compared to eyes with PNS. SAP–FDT difference of PSD value showed negative association with LCCI and LCD, which was significant in eyes with PNS compared to eyes with PFS. Glaucomatous eyes classified by the difference of the detected VF damage on FDT versus SAP showed different clinical features. Greater SAP–FDT difference was significantly associated with structural parameters such as LCD and LCCI. Less SAP–FDT difference was associated with presence of disc hemorrhage and lower deep layer peripepillary VD. There is possibility to use the difference of SAP and FDT to identify associated risk factors in glaucoma patients.

## Introduction

Glaucoma is characterized by progressive atrophy of the optic nerve head secondary to the loss of retinal ganglion cell (RGC) axons and nerve fibers. It shows a characteristic structural change and corresponding visual field (VF) defects with high elevation of intraocular pressure (IOP)^1^. In addition, previous studies suggested that the reduced ocular blood flow from a narrow central retinal arteriole with normal tension glaucoma (NTG) might induce glaucomatous damage, which supports the vascular theory of the pathogenesis of glaucomatous optic neuropathy. However, it remains unclear whether such association is a cause or a result of glaucomatous damage^2–4^.

Standard automated perimetry (SAP) using white stimulus on a white background is the most commonly used method for detection of glaucomatous functional damage. However, previous studies reported that SAP often detect visual field defects only after a substantial number of RGCs have been lost^5,6^. Frequency doubling technology (FDT) perimetry has been proposed to be more useful in detecting early glaucomatous functional damage than SAP^7,8^. It is based on the frequency doubling illusion, and each stimulus is a series of white and black bands flickering at 25 Hz^9^. Some studies claimed that FDT perimetry was similar to SAP in diagnostic performance of glaucoma. Spry et al.^10^ and Burgansky-Eliash et al.^11^ did not find superiority of FDT perimetry over SAP. However, FDT perimetry has been shown to be significantly better than SAP in discriminating glaucomatous and healthy eyes, and with shorter test duration and less variability in areas of low sensitivity than SAP^12,13^. FDT perimetry is thought to be mediated by a subset of the RGCs with large axonal diameter, called the M_6y_ ganglion cells, that project to the magnocellular (M) pathway^14^. These cells are sensitive to motion and contrast and are thought to be more vulnerable to mechanical glaucomatous damage than conventional perimetry^15,16^. On the other hand, SAP assesses the responses of both M and P cells, nonselectively, and both cells may be vulnerable to ischemic damage. We hypothesized that mechanical damage would be preferentially detected by FDT perimetry, while both mechanical and vascular damage would be detected by SAP. Therefore, we purposed to compare both perimetry and find out associated risk factors to the difference between FDT and SAP. The structural parameters may indicate mechanical damage of optic nerve head (ONH), and the vessel density may indicate vascular damage. We hypothesized that structural parameters may show stronger association with grater SAP–FDT difference, while vascular parameters with less SAP–FDT difference.

This study aimed to find out the significance of the difference between FDT perimetry and SAP in terms of the detected visual field (VF) damage, and evaluate associated factors to SAP–FDT difference in early glaucoma.

## Results

This study enrolled 96 eyes of 96 patients with early glaucoma who met the inclusion criteria and did not meet the exclusion criteria. Among 96 eyes, 41 (42.7%) eyes showed significant SAP–FDT difference more than 4 abnormal points and 55 (57.3%) eyes showed no SAP–FDT difference. In terms of the location of VF defect, 50 eyes had PNS and 46 eye had PFS on both VF tests. Interobserver agreement in terms of MvD identification was excellent (к = 0.908; 95% CI 0.901–0.914; *P* < 0.001). Table 1 shows the demographics of patients according to SAP–FDT difference. Eyes with SAP–FDT difference had significantly more frequent MvD (65.9%) compared to eyes without SAP–FDT difference (45.5%, *P* = 0.047). Additionally, eyes with SAP–FDT difference showed significantly greater LCD (538.68 ± 128.67 μm) and LCCI (11.10 ± 4.19) compared to eyes without SAP–FDT difference (475.73 ± 120.7 4 μm and 9.47 ± 3.50, respectively; all *P* < 0.05). Table 2 shows the demophgraphics and comparison between peripheral nasal step (PNS) and parafoveal scotoma (PFS) groups.

**Table 1:** Comparison of the demographics and test results between significant SAP–FDT difference.

	No SAP–FDT difference (n = 55)	Significant SAP–FDT difference (n = 41)	*P* value
Age (year)	54.33 ± 13.346	50.66 ± 12.222	0.171*
Sex (female, no (%))	29 (52.7%)	29 (70.7%)	0.074^†^
DM (no (%))	3 (54.5%)	2 (4.9%)	0.900^†^
HTN	9 (16.4%)	5 (12.2%)	0.567^†^
History of migraine (no (%))	10 (18.2%)	8 (19.5%)	0.869^†^
CCT (μm)	533.73 ± 41.072	530.83 ± 37.852	0.725*
Axial length (mm)	24.95 ± 1.53	25.33 ± 1.16	0.180*
Intraocular pressure (mmHg)	14.56 ± 3.14	14.690 ± 2.60	0.834*
Mean deviation, SAP (− dB)	− 2.94 ± 1.90	− 2.80 ± 1.60	0.204*
Pattern standard deviation, SAP (− dB)	5.49 ± 2.511	5.07 ± 2.479	0.409*
Mean deviation, FDT (− dB)	− 6.51 ± 3.85	− 7.18 ± 3.49	0.400*
Pattern standard deviation, SAP (− dB)	5.64 ± 1.72	6.19 ± 1.48	0.105*
Average RNFL thickness (μm)	74.75 ± 10.34	77.76 ± 8.19	0.127*
Presence of MvD (no (%))	25 (45.5%)	27 (65.9%)	**0.047**^†^
Presence of disc hemorrhage (no (%))	26 (47.3%)	14 (34.1%)	0.197^†^
Superficial PPVD	45.56 ± 3.58	46.47 ± 2.60	0.149*
Deep PPVD	50.17 ± 8.63	49.35 ± 8.50	0.642*
LCD (μm)	475.73 ± 120.74	538.68 ± 128.67	0.017*
LCCI	9.47 ± 3.50	11.10 ± 4.19	0.040*

The significant SAP–FDT difference is defined as depressed points of P < 5% and P < 1% on the PSD plot greater than 4 points.

Mean values are presented with standard deviations.

*No* number, *DM* diabetes, *HTN* hypertension, *CCT* central corneal thickness, *RNFL* retinal nerve fiber layer, *MvD* microvascular dropout; *PPVD* peripapillary vessel density, *LCD* lamina cribrosa depth, *LCCI* lamina cribrosa curvature index.

Bold font indicates significant P values (P < 0.05).

*Student’s t-test.

^†^Chi-squared test.

**Table 2:** Comparison of the demographics and test results between peripheral nasal step and parafoveal scotoma.

	Peripheral nasal step (n = 50)	Parafoveal scotoma (n = 46)	*P* value
Age (year)	51.06 ± 13.704	5461 ± 11.930	0.181*
Sex (female, no (%))	30 (60.0%)	28 (60.9%)	0.931^†^
History of migraine (no (%))	6 (12.0%)	12 (26.1%)	0.077^†^
CCT (μm)	530.80 ± 46.95	534.33 ± 29.92	0.659*
Axial length (mm)	23.64 ± 5.20	23.96 ± 6.21	0.783*
Intraocular pressure (mmHg)	14.66 ± 3.264	14.57 ± 2.587	0.887*
Mean deviation, SAP (− dB)	− 3.01 ± 1.856	− 2.74 ± 1.682	0.458*
Pattern standard deviation, SAP (− dB)	4.71 ± 2.472	5.96 ± 2.376	0.013*
Mean deviation, FDT (− dB)	− 7.20 ± 3.61	− 6.40 ± 3.79	0.289*
Pattern standard deviation, FDT (-dB)	6.23 ± 1.79	5.49 ± 1.36	0.025*
Average RNFL thickness (μm)	75.56 ± 10.065	76.54 ± 9.050	0.617*
Presence of MvD (no (%))	25 (50.0%)	27 (58.7%)	0.393^†^
Presence of disc hemorrhage (no (%))	22 (44.0%)	18 (39.1%)	0.629^†^
Superficial PPVD	45.27 ± 2.85	46.69 ± 3.46	0.030*
Deep PPVD	51.07 ± 8.754	48.46 ± 8.169	0.136*
LCD (μm)	495.42 ± 130.985	510.43 ± 124.407	0.567*
LCCI	10.83 ± 4.080	9.45 ± 3.548	0.082*

Mean values are presented with standard deviations.

*No* number, *CCT* central corneal thickness, *RNFL* retinal nerve fiber layer, *MvD* microvascular dropout, *PPVD* peripapillary vessel density, *LCD* lamina cribrosa depth, *LCCI* lamina cribrosa curvature index.

Bold font indicates significant P values (P < 0.05).

*Student’s t-test.

^†^Chi-squared test.

To find out associated factors related to the presence of SAP–FDT difference, logistic regression analysis was performed (Table 3). Female sex, presence of MvD, no disc hemorrhage, and greater LCD showed significant association with SAP–FDT difference in both univariate and multivariate analysis (all *P* < 0.05, respectively).

**Table 3:** Factors associated with the SAP–FDT difference using logistic regression analysis.

	Univariate β (95% CI)	*P* value	Multivariate β (95% CI)	*P* value
Sex (female, %)	1.018 (0.898–8.534)	**0.076**	1.190 (1.235–8.754)	**0.017**
IOP (mmHg)	0.052 (0.916–1.212)	0.466		
Average RNFL thickness (μm)	0.037 (0.977–1.101)	1.037		
Presence of MvD (%)	1.236 (1.103–10.751)	**0.033**	1.086 (1.165–7.533)	**0.023**
Presence of disc hemorrhage (%)	− 1.461 (0.076–0.709)	**0.010**	− 1.029 (0.138–0.927)	**0.034**
Mean deviation, SAP (− dB)	− 0.039 (0.682–1.357)	0.824		
Pattern standard deviation, SAP (− dB)	− 0.086 (0.716–1.176)	0.496		
Superfical PPVD	0.001 (0.999–1.002)	0.248		
Deep PPVD	− 0.019 (0.919–1.047)	0.560		
LCD	0.004 (1.000–1.009)	**0.077**	0.005 (1.001–1.009)	**0.007**
LCCI	0.114 (0.975–1.289)	0.108		

*CI* confidence interval, *IOP* intraocular pressure, *RNFL* retinal nerve fiber layer, *MvD* microvascular dropout, *SAP* standard automated perimetry, *PPVD* peripapillary vessel density, *LCD* lamina cribrosa depth, *LCCI* lamina cribrosa curvature index.

Variables with P < 0.10 were included in the multivariate analysis.

Factors with statistical significance are shown in bold.

**Figure 1:**
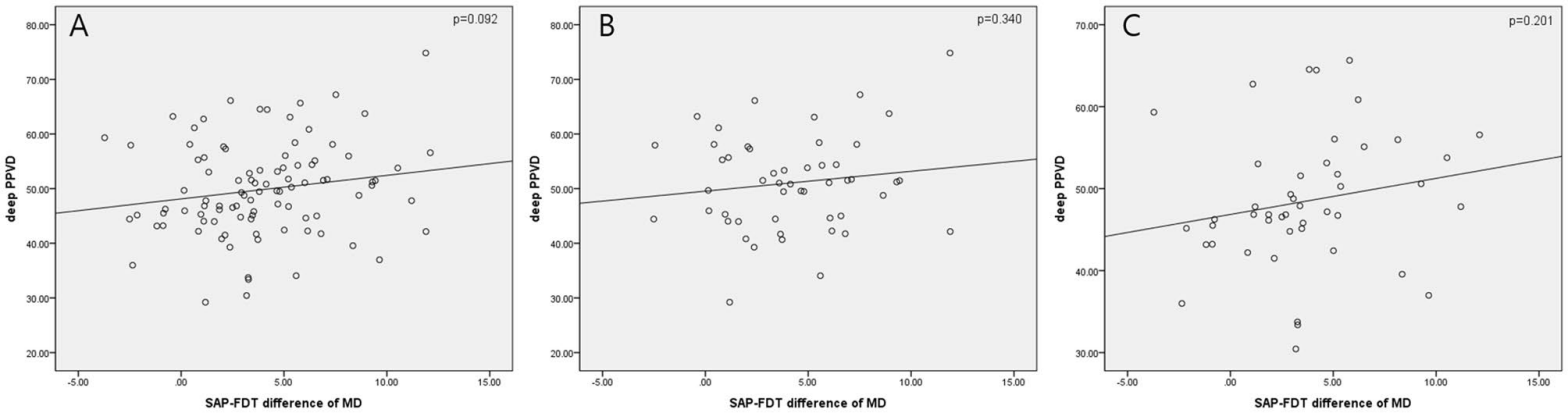
Scatterplot between SAP–FDT difference of MD and deep layer of peripapillary vessel density. Difference of MD between VFs tends to decrease, as VD decreases in whole group (**A**), in peripheral nasal step (PNS) group (**B**), and in parafoveal scotoma group (PFS) (**C**), without statistical significance.

**Figure 2:**
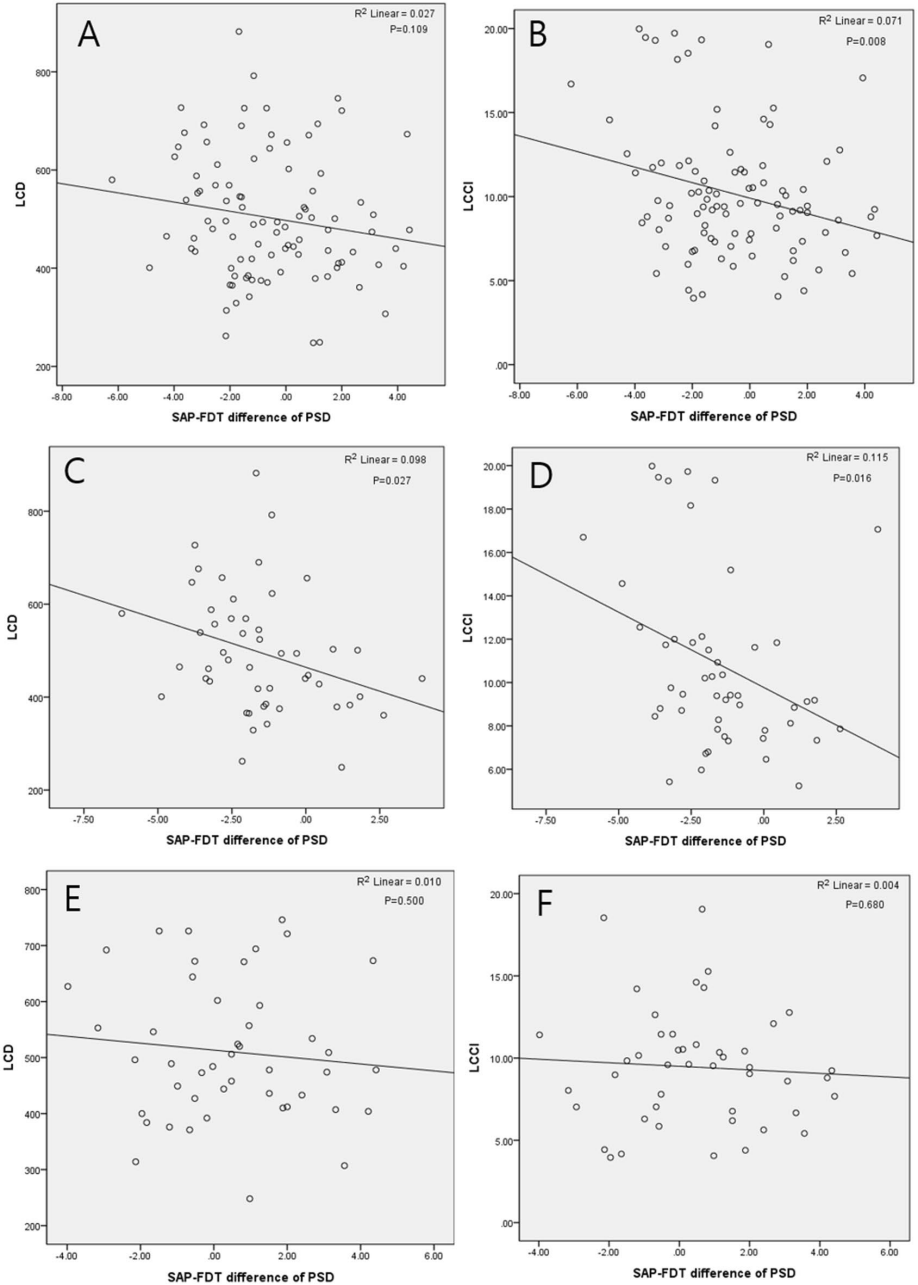
Scatterplot between SAP–FDT difference of pattern standard deviation (PSD) and posterior deformation of lamina cribrosa. In whole group, SAP–FDT difference of PSD and lamina cribrosa depth (LCD) or lamina cribrosa curvature index (LCCI) showed negative correlation (**A,B**). As the SAP–FDT difference of PSD gets greater, there was increase in LCD and LCCI with statistical significance in peripheral nasal step group (**C,D**). This trend was not observed in parafoveal scotoma group (**E,F**). Linear regression analysis was used.

The scatterplot between SAP–FDT difference of MD and deep peripapillary VD showed positive trend in Fig. 1. As the SAP–FDT difference of MD gets smaller, there was decrease in deep peripapillary VD, although there was no statistical significance (*P* = 0.092) (Fig. 1A). This trend was observed in both PNS and PFS groups without significance (*P* = 0.340, *P* = 0.201) (Fig. 1B,C) The scatterplot between SAP–FDT difference of PSD and LCD or LCCI showed negative correlation in Fig. 2. As the SAP–FDT difference of PSD gets greater, there was significant increase in LCCI, not in LCD (*P* = 0.008, *P* = 0.109) (Fig. 2A,B). Both LCD and LCCI showed negative correlations in PNS group (*P* = 0.027; *P* = 0.016) (Fig. 2C,D), but not in PFS group (*P* = 0.500; *P* = 0.680) (Fig. 2E,F).

To find out associated factors to SAP–FDT difference in early glaucomatous eyes with PNS and PFS, logistic regression analyses for each group were performed (Tables 4, 5). Presence of MvD, no disc hemorrhage, and greater LCD were significantly associated factors with SAP–FDT difference in PFS group in multivariate analysis (all P < 0.05). Whereas, nothing was associated with SAP–FDT difference in PNS group.

**Table 4:** Factors associated with the SAP–FDT difference using logistic regression analysis in peripheral nasal step.

	Univariate β (95% CI)	*P* value	Multivariate β (95% CI)	*P* value
Sex (female, %)	1.118 (0.655–14.304)	0.155		
IOP (mmHg)	0.037 (0.847–1.272)	0.722		
Average RNFL thickness (μm)	0.041 (0.959–1.133)	0.333		
Presence of MvD (%)	1.109 (0.477–19.273)	0.240		
Presence of disc hemorrhage (%)	− 0.534 (0.113–3.030)	0.524		
Mean deviation, SAP (− dB)	0.065 (0.584–1.948)	0.833		
Pattern standard deviation, SAP (− dB)	− 0.234 (0.511–1.226)	0.295		
Superfical PPVD	0.001 (0.999–1.003)	0.491		
Deep PPVD	− 0.003 (0.907–1.095)	0.948		
LCD	− 0.002 (0.991–1.005)	0.561		
LCCI	0.133 (0.477–19.273)	0.240		

*CI* confidence interval, *IOP* intraocular pressure, *RNFL* retinal nerve fiber layer, *MvD* microvascular dropout, *SAP* standard automated perimetry, *PPVD* peripapillary vessel density, *LCD* lamina cribrosa depth, *LCCI* lamina cribrosa curvature index.

Variables with P < 0.10 were included in the multivariate analysis.

**Table 5:** Factors associated with SAP–FDT difference using logistic regression analysis in parafoveal scotoma.

	Univariate β (95% CI)	*P* value	Multivariate β (95% CI)	*P* value
Sex (female, %)	2.488 (0.661–21.92)	**0.093**	1.730 (0.908–35.058)	0.063
IOP (mmHg)	0.151 (0.845–1.600)	0.354		
Average RNFL thickness (μm)	0.127 (0.938–1.375)	0.192		
Presence of MvD (%)	3.079 (0.815–57.999)	**0.066**	1.774 (1.085–32.043)	**0.040**
Presence of disc hemorrhage (%)	− 3.273 (0.002–0.692)	**0.027**	− 1.761 (0.033–0.883)	**0.035**
Mean deviation, SAP (− dB)	− 0.778 (0.173–1.219)	0.118		
Pattern standard deviation, SAP (− dB)	− 0.036 (0.597–1.558)	0.882		
Superfical PPVD	0.001 (0.998–1.004)	0.472		
Deep PPVD	− 0.116 (0.762–1.041)	0.145		
LCD	0.017 (1.004–1.031)	**0.013**	0.012 (1.004–1.020)	**0.004**
LCCI	0.182 (0.879–1.638)	0.250		

*CI* confidence interval, *IOP* intraocular pressure, *RNFL* retinal nerve fiber layer, *MvD* microvascular dropout, *SAP* standard automated perimetry, *PPVD* peripapillary vessel density, *LCD* lamina cribrosa depth, *LCCI* lamina cribrosa curvature index.

Variables with P < 0.10 were included in the multivariate analysis.

Factors with statistical significance are shown in bold.

**Figure 3:**
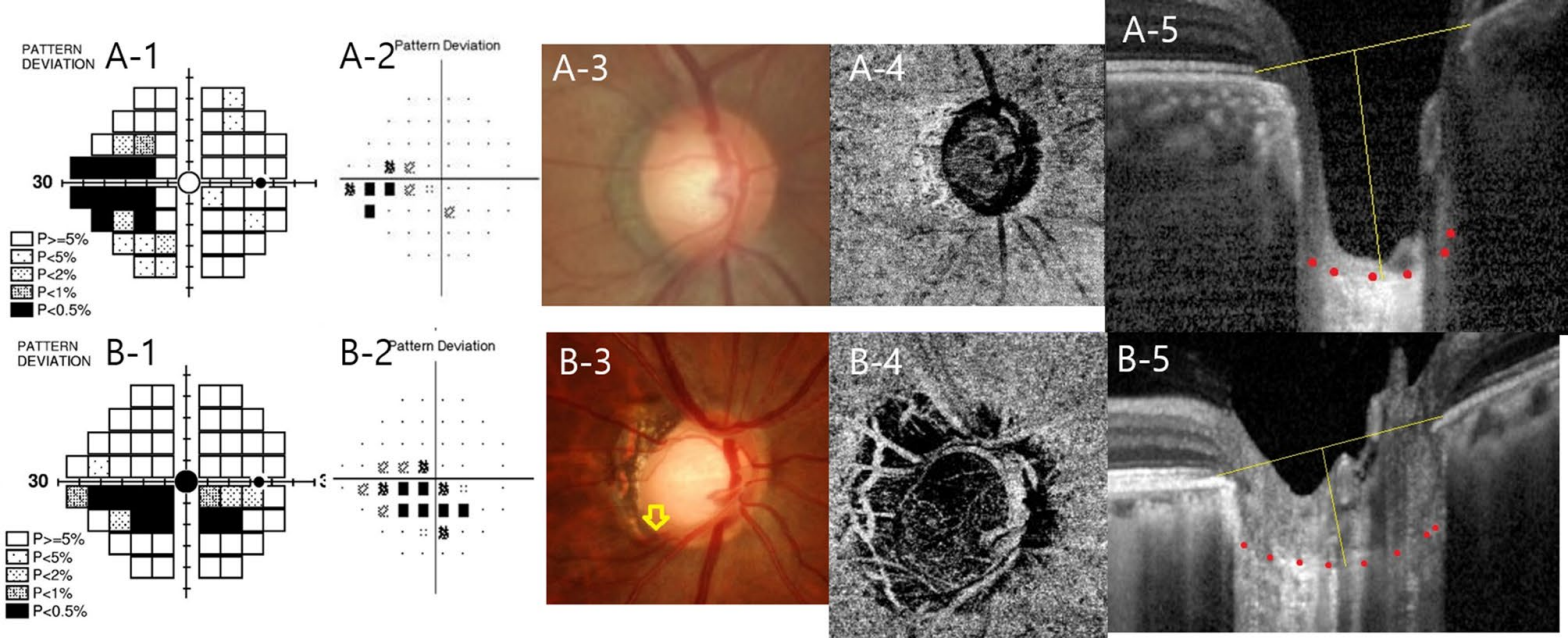
Representative cases of SAP–FDT difference in peripheral nasal step (PNS) and parafoveal scotoma (PFS) in early stage of open-angle glaucoma. (**A**) A 51-year-old woman with PNS presented with great SAP–FDT difference. (**A-1,A-2**). Lamina cribrosa depth (LCD) of 727 μm and lamina cribrosa curvature index (LCCI) of 19.21 was larger (**A-5**) than those of eye with PFS (**B-5**). (**B**) A 50-year-old man with PFS presented with 14 abnormal points in FDT and 16 abnormal points in SAP (**B-1,B-2**). Fundus photography showed disc hemorrhage (yellow arrow) (**B-3**). Peripapillary choroidal VD of 53.80% was low (**B-4**).

As shown in representative cases in Fig. 3, eye with significant SAP–FDT difference shows PNS defects on both SAP and FDT that is greater on FDT. This patient shows deep LCD and greater LCCI on EDI image of Spectralis OCT (Fig. 3A). In contrast, eye without SAP–FDT difference shows PFS defects on both SAP and FDT that is similar on both VFs. This patient did have LC changes similar to the previous case, however, showed disc hemorrhage and had lower VD within the PPA region (Fig. 3B).

## Discussion

This study evaluated associated factors to the difference between FDT perimetry and SAP in eyes with early stage of glaucoma. In eyes with VF defect largely detected on FDT compared to SAP had features such as deep LCD and greater LCCI that may represent structural parameters of the ONH. Also, presence of MvD was significantly frequent in eyes with SAP–FDT difference that may indicate LC changes accompanying VD changes at the deep layer of the OCT-A. In contrast, eyes with no SAP–FDT difference showed significant association with the presence of disc hemorrhage, and tended to have lower deep peripapillary VD on OCT-A. These indicate that comparing FDT perimetry and SAP could give us clues whether structural damage or vascular damage is the contributing mechanism of RGC loss in a particular patient.

The transfer of visual information from the eye to visual cortex involves the relay of signals which comprise six layers through the dorsal lateral geniculate nucleus (LGN) of the thalamus. Function-specific RGC terminals target anatomically distinct readily identifiable LGN layers. The functional channels are composed of magnocellular (M), parvocellular (P), and koniocellular (K) pathways^17^. The bigger M cells are sensitive to higher temporal frequency and receive their major retinal input from the parasol RGCs. The smaller P neurons receive their retinal input from the midget RGCs, and are sensitive to higher spatial frequencies and convey red-green color information^18^. Some previous studies demonstrated that there does not appear to be any vulnerability of a specific type nor any preferential loss of RGCs in experimental glaucoma^19–21^. It was suggested that eyes with Leber hereditary optic neuropathy, primary mitochondrial DNA disorder causing metabolic disturbance, even had loss of the midget, parasol and bistratified RGCs associated with all three principal visual pathways^22^. However, Quigley et al.^23^ suggested that RGCs with larger axonal diameter may undergo earlier loss than others by glaucomatous damage, although no fiber size is spared from damage. This preferential loss of large fibers appears to be due to a higher proportion of the fibers in the inferior and superior poles and an inherent susceptibility to injury by glaucoma. Also, IOP elevation appears to have a more profound degenerative effect on the M-cell than on the P-cell regions in monkey eyes^24^. Furthermore, models of glaucoma induced in primates, cats, and mice also show selective effects on RGC types, particularly OFF RGCs. The mechanisms may be differences in the expression of calcium-permeable receptors, the relative proximity of RGCs, and their dendrites to blood supply in the inner plexiform layer^25^. Therefore, these studies suggest that mechanical damage would preferably result earlier loss of M-cell than P-cell region in early glaucomatous damage. Since these RGCs are located in the areas that suffer most rapid loss of fibers, especially in the periphery of optic nerve, this damage would lead to early PNS defects^23^. This may in part explain why structural parameters were significantly associated with SAP–FDT difference in eyes with PNS.

As mentioned above, FDT perimetry is thought to detect more sensitively to mechanical glaucomatous damage than SAP in that frequency-doubling stimulus is preferentially stimulating M cells versus parvocellular (P) ganglion cells^26^. In this study, we evaluated LCD and LCCI for the indicator of structural changes of the ONH. Eyes with greater SAP–FDT difference had significantly greater LCD and LCCI than the group without SAP–FDT difference. The greater SAP–FDT difference of PSD showed the greater degree of LCD and LCCI. This tendency was significant in eyes with PNS. As VF defect located in PNS may result from nerve fiber bundle defect where the LC is vulnerable to mechanical damage, we may assume that VF defect on FDT could represent damage with mechanical stress to the LC^27^.

Eyes with less SAP–FDT difference of MD had a tendency toward lower peripapillary choroidal VD. The VD of the deep layer around optic disc affects ocular microcirculation^28^, as peripapillary choroidal microvasculature is supplied by the short posterior ciliary arteries^29^. Presence of disc hemorrhage was associated with less SAP–FDT difference. These results were prominent in isolated PFS group. (Tables 3, 5) Among the eyes with disc hemorrhage, disc hemorrhage in the inferotemporal region was the most common (32 eyes), followed by superotemporal, both superotemporal and inferotemporal, and inferonasal region. Disc hemorrhage has been considered a sign of ischemic optic nerve damage due to vascular insufficiency^30–32^. Previous studies demonstrated that pathologic mechanisms of PFS involves vascular factors unlike peripheral VF loss^33,34^. This study suggests that eyes with ischemic damage in addition to ONH morphologic change, has a tendency to show similar VF defect on both FDT and SAP. Enroth-Cugell et al.^35^ reported that hypoxia disrupts some early stage of retinal processing, as well as, hypoxia acts rather diffusely on all types of RGCs. Signals could be carried through horizontal cells located in an anoxic area of retina after transmission of signals from receptors to horizontal cells in the region was abolished^36^. It was suggested that ON pathway, which is mostly P-cell, would be more metabolically active in primates and metabolic active RGCs could be more vulnerable to ischemic damage^25^. This is why PFS is presented in glaucomatous eyes with vascular insufficiency since metabolic demand of the central region of the retina is high. RGC loss involving both P- and M-cells may indicate that vascular mechanism is involved in the earlier stage of glaucoma process in those patients.

Recent studies reported that MvD is associated with structural changes in ONH structures including focal LC defects, thinner choroidal thickness, and beta or gamma PPA^37–39^. Shin et al.^40^ reported that peripapillary choroidal MvD was associated with the presence of scleral deformation related to corresponding RNFL defects. They hypothesized that LC deformation may account for the generation of dropout-causing closure of choriocapillaries passing through the sclera to supply the ONH. Then, the closed short posterior ciliary artery reduces blood supply to the ONH. The present study also supports that MvD may derive from mechanical damage of ONH; therefore, it was associated with significant SAP–FDT difference in eyes with early glaucoma. When MvD is present, it has been reported that those eyes frequently present with PFS^41^. Since the presence of MvD may be an indicator of vascular insufficiency in the deep ONH structures, damage in the central VF region where the metabolic demand is high may occur earlier than damage outside the 10° region.

This study has several limitations. First, small sample size may limit generalization of the present findings. Second, since the study measurements involved a single Korean ethnic group with a higher prevalence of normal-tension glaucoma, the study results may be different in other populations with a greater prevalence of high-tension glaucoma where mechanical stress might be more prominent. Further studies including more subjects of different ethnic backgrounds may yield additional information by comparing FDT and SAP. Third, early glaucoma patients with MD on SAP better than − 6.0 dB were included in the present study. The SAP–FDT difference may be found only in the early stage of the disease and usually the results of SAP and FDT gets similar as the disease progresses. Therefore, our findings may be applicable in early glaucoma. Fourth, retinal vessel signals evident on en face, deep-layer of OCT-A images render it difficult to precisely measure the choroidal VD. However, previous studies^42,43^ have found that the repeatability and reproducibility of measurements were good in the deep layer, as it was confirmed by two observers. Finally, LC surface reference line should be set from the LC insertion points to allow the precise quantification of LC curvature. However, only the LC within the BMO width was included for measuring the LC curvature in the present study, because since the LC was often not visible outside of this region. Nevertheless, Lee et al.^44^ demonstrated that the LCCI measured from the whole LC, between the LC insertions, was comparable with that measured on the LC within BMO in eyes with visible LC up to LC insertion.

In conclusion, evaluating the difference in the VF defects on FDT and SAP could help predicting associated factors to the glaucomatous damage. In eyes with SAP–FDT difference, which means larger VF defects on FDT, seems to be associated with structural parameters such as LC parameters and presence of MvD that may indicated mechanical damage to the ONH. In contrast, eyes with no SAP–FDT difference, which means similar VF defects on FDT and SAP, seems to be associated with disc hemorrhage and lower deep peripapillary VD that indicates vascular damage. In clinical practice, it may help to predict associated risks and make plans for glaucoma evaluation and treatment by comparing FDT and SAP. Further studies are needed to confirm the clinical significance.

## Methods

The medical records of glaucoma patients were obtained from the database of the Catholic Medical Center Glaucoma Progression Study (CMC-GPS) which commenced in 2009 at Seoul St. Mary’s Hospital, Seoul, South Korea. The work was approved by our institutional review board of Seoul St. Mary’s Hospital and we followed all relevant tenets of the Declaration of Helsinki. We enrolled all consecutive eligible patients who were willing to participate, and all gave written informed consent.

All glaucoma patients enrolled in the CMC-GPS underwent a complete ophthalmic examination, including a review of medical history, measurement of best-corrected visual acuity, refraction assessment, slit-lamp biomicroscopy, gonioscopy, Goldmann applanation tonometry, measurement of central corneal thickness via ultrasound pachymetry (Tomey Corp., Nagoya, Japan), measurement of axial length with ocular biometry (IOL Master; Carl Zeiss Meditec, Dublin, CA, USA), dilated stereoscopic examination of the optic disc, red-free fundus photography (Canon, Tokyo, Japan), and Cirrus optical coherence tomography (OCT; Carl Zeiss Meditec). Starting from 2017, patient underwent additional OCT-A (DRI OCT Triton; Topcon, Tokyo, Japan) examinations. All disc hemorrhage detected on the color disc and fundus photography during follow-up were recorded.

Open-angle glaucoma was defined by the presence of a glaucomatous optic disc (exhibiting diffuse or localized rim thinning, a notch in the rim, or a vertical cup-to-disc ratio ≥ 0.2 than that of the other eye); a VF finding consistent with glaucoma (a cluster of ≥ 3 non-edge points on the pattern deviation plot with a probability of < 5% of the normal population, with one of these points having a probability of < 1%), a pattern standard deviation (PSD) with a *P*-value < 5%, or a Glaucoma Hemifield Test result consistently outside the normal limits on two VF examinations either on FDT perimetry or SAP as confirmed by two glaucoma specialists (H.Y.P. and C.K.P.); and an open angle evident on gonioscopy. Additional inclusions were: a best-corrected visual acuity ≥ 20/40, a spherical refraction within ± 6.0 diopters (D), a cylinder correction within ± 3.0 D, and mean deviation (MD) better than − 6 dB on SAP. The exclusion criteria were: a history of any retinal disease, including diabetic or hypertensive retinopathy; a history of eye trauma or surgery with the exception of uncomplicated cataract surgery; any optic nerve disease apart from glaucoma; and a history of systemic or neurological diseases that might affect the VF. If both eyes of an enrolled patient met all inclusion and exclusion criteria, one eye was randomly chosen for study.

### Visual field testing

All subjects underwent SAP using 24-2 SITA standard programs with a Humphrey field analyzer II 750i (Carl Zeiss Meditec, Dublin, CA). Goldmann size III targets with diameters of 0.43° were presented. FDT perimetry was performed using the 24-2 program with 5° stimuli, spatial frequency of 0.5 cycles/deg, and temporal frequency of 18 Hz with the FDT Humphrey Matrix (Carl Zeiss Meditec). All the VF tests were done within no more than 3 months. The sequences of VF test were not standardized. Reliable tests were defined as < 15% fixation losses, false positives, or false negatives.

**Figure 4:**
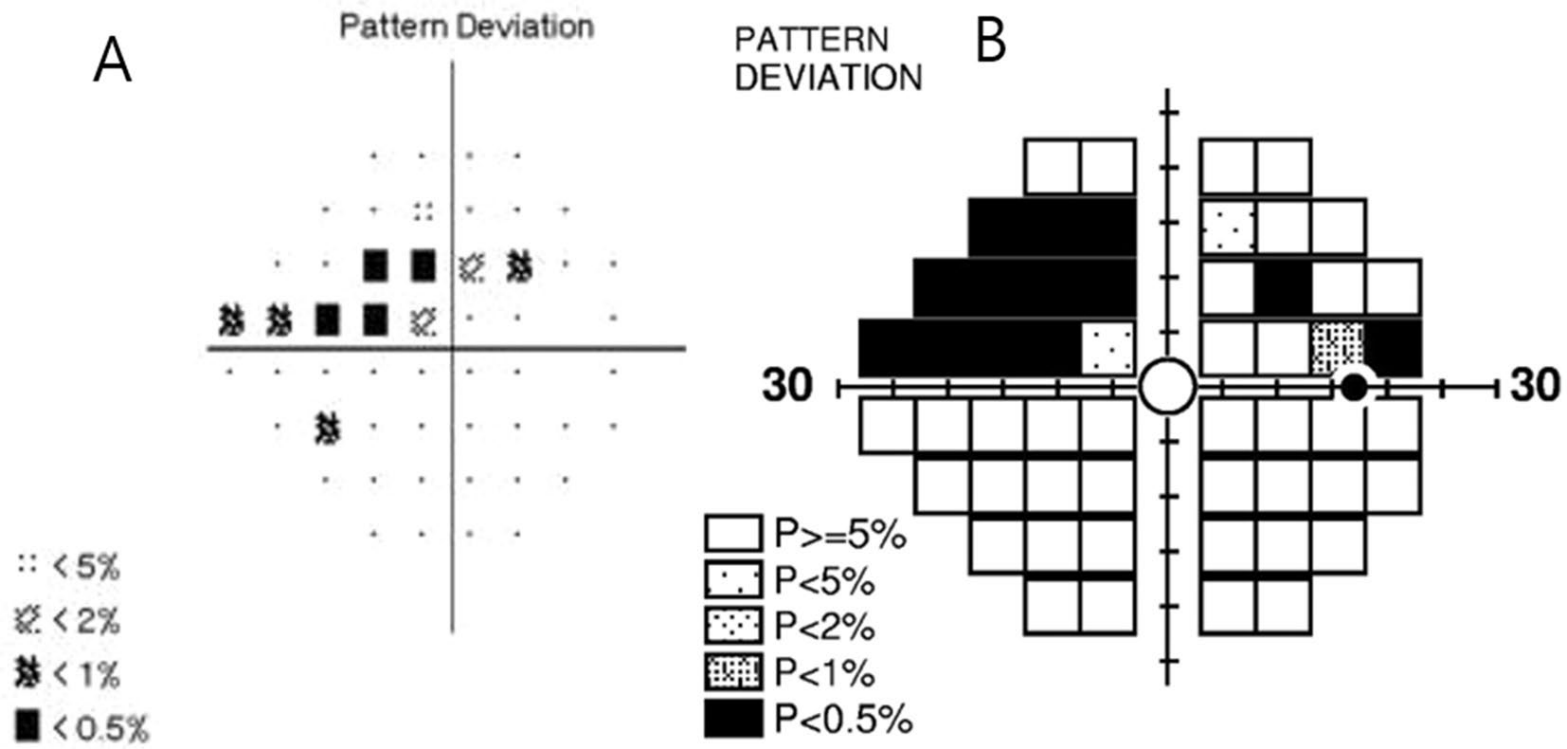
Definition of significant SAP–FDT difference. There were 10 depressed points of P < 5% and P < 1% on pattern deviation plot of SAP (**A**). The depressed points of P < 5% and P < 1% on pattern deviation plot of FDT were 15 (**B**). We subtracted the abnormal points of FDT from those of SAP, and it was 5 points. This eye was significant SAP–FDT difference, as significant SAP–FDT difference was defined as greater than 4 abnormal points.

To define SAP–FDT difference, we used MD and PSD of both SAP and FDT perimetry. We subtracted MD and PSD of FDT from those of SAP, respectively. Additionally, we compared the number of abnormal points depressed as P < 5% and P < 1% on the pattern deviation plot on both SAP and FDP perimetry. We compared the number of abnormal points of FDT from those of SAP, and eyes were classified to have significant SAP–FDT difference when the number of abnormal points were more than 4 points on FDT (Fig. 4).

Among all patients, eyes with peripheral nasal step (PNS) and parafoveal scotoma (PFS) were determined based on pattern deviation probability plots of FDT perimetry. PFS subjects had an isolated glaucomatous VF defect within twelve points of a central 10° radius. PNS subjects had an isolated glaucomatous VF damage within the nasal periphery outside 10° fixation. Patients with abnormal points in both regions were excluded.

### Optic nerve head parameters

According to the mechanical theory of glaucoma, posterior deformation of the LC is a principle pathologic event^45^. A potential indicator of the LC morphology is the LC depth (LCD), because LCD is correlated with the magnitude of the posterior LC deformation^46,47^. The efficacy in discriminating between glaucomatous eye and healthy eyes was better for LC curvature index (LCCI) than, therefore LC curvature may be a parameter for LC morphology^48,49^.

Heidelberg spectralis OCT provides up to 40,000 A-scans/s with a depth resolution of 7 μm in tissues, and a transverse resolution of 14 μm in images of ocular microstructures. Enhanced depth imaging (EDI)-OCT B-scans around optic nerve head (ONH) (6 mm optic cube scans) were obtained using the spectralis OCT. Each section was obtained using eye tracking and incorporated an average of at least 35 OCT frames. Images with a quality score > 15 were obtained (~ 65–70 sections per eye). LCD and LCCI were measured based on the average images, as presented in previous studies^48,50^. Measurements were done using caliper function of the OCT software by an observer (KSA). The measurements were performed in the superior mid-peripheral, center, and inferior mid-peripheral regions, which were scanned throughout the ONH.

LCD was determined by measuring the distance from a line connecting the edges of Bruch’s membrane opening (BMO), reference line, perpendicular to the level of the anterior LC surface. The average of three measurements of each region, as explained above was used to derive the LCD.

For measurement of LCCI, a reference line (LC surface reference line) was set in each B-scan by connecting the two points on the anterior LC surface that met with the lines drawn from each Bruch’s membrane termination point perpendicularly to the BMO reference line. The length of reference line was defined as width (*W*). The LCD was defined as the maximum depth from the reference line to the anterior LC surface. Then, LCCI was calculated as (LCD/*W*) × 100. LCCI and *W* were measured at three regions each, and then calculated LCCI respectively. The average of three LCCIs was used to derive the LCCI.

### Peripapillary vessel density

Swept-source optical coherence tomography (SS-OCT) was performed using the deep-range imaging (DRI)-OCT system (Topcon, Tokyo, Japan) with a wavelength of 1050 nm and scan speed of 100,000 A-scans per second, using Topcon OCT-A ratio analysis algorithm. Optical coherence tomography angiography of the DRI OCT generated en face images via automated layer segmentation around the optic nerve head into 4 layers. We chose the radial peripapillary capillary (RPC) mode, which estimate a 70 µm thick layer below the internal limiting membrane (ILM), to assess the superfacial layer. The deep layer peripapillary choroidal microvasculature in the relevant region was evaluated using en face images generated via automated layer segmentation of signals from the retinal pigment epithelium. Clear images with quality score over 30 were analyzed. To measure choroidal VD, the boundaries of the optic disc and β-zone peripapillary atrophy (PPA) were delineated using ImageJ software (National Institutes of Health). Eyes without β-zone PPA were excluded. An 8-bit binary slab was created according to the mean threshold algorithm of ImageJ, which automatically computed the threshold value as the mean of the local grayscale distribution. After assigning white pixels as vessels and black pixels as background, parapapillary choroidal VD was defined as a percentage of vessel pixels within the β-zone PPA region relative to the total area of the β-zone PPA.

**Figure 5:**
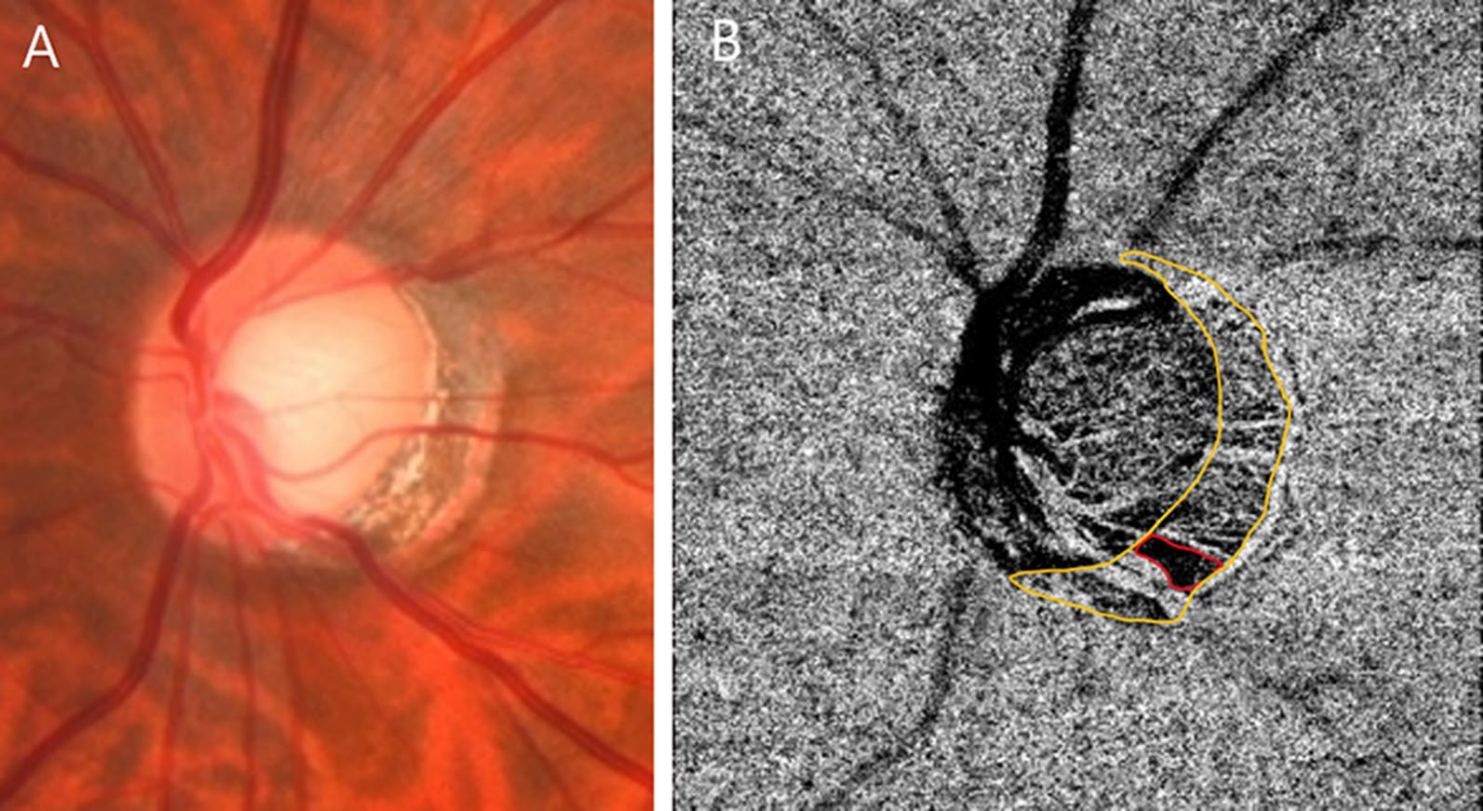
Definitiion of microvascular dropout (MvD). MvD was defined as focal, sectoral complete loss of choriocapillaris within the visible microvascular network. MvD in the left eye. Sectoral complete loss of choriocapillaris (red line) in peripapillay atrophy (yellow line).

The deep-layer parapapillary microvasculature in the relevant region was evaluated using *en face* images generated by automated layer segmentation of signals from the retinal pigment epithelium, which extended to the outer scleral border Microvascular dropout (MvD) was defined as focal, sectoral complete loss of choriocapillaris within the visible microvascular network. MvD was identified based on a dropout width > 2-fold that of the visible juxtapapillary microvessels (Fig. 5). Two independent observers (H.Y.P and S.A.K.) blinded to the clinical data identified MvD. Disagreements were resolved by a third observer (C.K.P.). Only clear images (quality scores > 30 and no motion blurring) were analyzed.

### Statistical analysis

Interobserver difference in MvD identifications were evaluated using к coefficients. Differences in continuous variables between two groups were compared using independent *t*-test, while categorial variables were compared using χ^2^ test. To determine the clinical variables associated with the difference of SAP and FDT univariate and multivariate logistic regression analyses were performed. The significant SAP–FDT that is more than 4 abnormal points on pattern deviation plot on FDT than SAP was anaylzed as dependent variable. Variables with a *P* value less than 0.10 in univariate analysis were entered into multivariate analysis. Relationships between difference of MD and PSD between two VFs, deep peripapillary VD, and optic nerve head parameters, such as LCD, LCCI, and LC thickness were analyzed in linear regression analysis. *P* value < 0.05 indicated statistical significance. All statistical analysis was performed using SPSS software version 21.0 (IBM cop., Armonk, NY, USA).

## References

[1] Weinreb RN, Khaw PT (2004). Primary open-angle glaucoma. Lancet.

[2] Wang S, Xu L, Wang Y, Wang Y, Jonas JB (2007). Retinal vessel diameter in normal and glaucomatous eyes: The Beijing eye study. Clin. Exp. Ophthalmol..

[3] Amerasinghe N (2008). Evidence of retinal vascular narrowing in glaucomatous eyes in an Asian population. Investig. Ophthalmol. Vis. Sci..

[4] Lee JY, Yoo C, Park JH, Kim YY (2012). Retinal vessel diameter in young patients with open-angle glaucoma: Comparison between high-tension and normal-tension glaucoma. Acta Ophthalmol..

[5] Harwerth RS, Quigley HA (2006). Visual field defects and retinal ganglion cell losses in patients with glaucoma. Arch. Ophthalmol..

[6] Quigley HA, Dunkelberger GR, Green WR (1989). Retinal ganglion cell atrophy correlated with automated perimetry in human eyes with glaucoma. Am. J. Ophthalmol..

[7] Landers J, Goldberg I, Graham S (2000). A comparison of short wavelength automated perimetry with frequency doubling perimetry for the early detection of visual field loss in ocular hypertension. Clin. Exp. Ophthalmol..

[8] Sample PA, Bosworth CF, Blumenthal EZ, Girkin C, Weinreb RN (2000). Visual function-specific perimetry for indirect comparison of different ganglion cell populations in glaucoma. Investig. Ophthalmol. Vis. Sci..

[9] Cello KE, Nelson-Quigg JM, Johnson CA (2000). Frequency doubling technology perimetry for detection of glaucomatous visual field loss. Am. J. Ophthalmol..

[10] Spry PG, Hussin HM, Sparrow JM (2005). Clinical evaluation of frequency doubling technology perimetry using the Humphrey matrix 24–2 threshold strategy. Br. J. Ophthalmol..

[11] Burgansky-Eliash Z (2007). Glaucoma detection with matrix and standard achromatic perimetry. Br. J. Ophthalmol..

[12] Liu S (2011). Comparison of standard automated perimetry, frequency-doubling technology perimetry, and short-wavelength automated perimetry for detection of glaucoma. Investig. Ophthalmol. Vis. Sci..

[13] Artes PH, Hutchison DM, Nicolela MT, LeBlanc RP, Chauhan BC (2005). Threshold and variability properties of matrix frequency-doubling technology and standard automated perimetry in glaucoma. Investig. Ophthalmol. Vis. Sci..

[14] Maddess T (1999). Testing for glaucoma with the spatial frequency doubling illusion. Vis. Res..

[15] Quigley HA (1999). Neuronal death in glaucoma. Prog. Retin Eye Res..

[16] Read RM, Spaeth GL (1974). The practical clinical appraisal of the optic disc in glaucoma: The natural history of cup progression and some specific disc-field correlations. Trans. Am. Acad. Ophthalmol. Otolaryngol..

[17] Hendry SH, Calkins DJ (1998). Neuronal chemistry and functional organization in the primate visual system. Trends Neurosci..

[18] Perry VH, Oehler R, Cowey A (1984). Retinal ganglion cells that project to the dorsal lateral geniculate nucleus in the macaque monkey. Neuroscience.

[19] Harwerth RS (2002). Visual field defects and neural losses from experimental glaucoma. Prog. Retin. Eye Res..

[20] Morgan JE (2002). Retinal ganglion cell shrinkage in glaucoma. J. Glaucoma.

[21] Martin L, Wanger P, Vancea L, Göthlin B (2003). Concordance of high-pass resolution perimetry and frequency-doubling technology perimetry results in glaucoma: No support for selective ganglion cell damage. J. Glaucoma.

[22] Majander A (2017). The pattern of retinal ganglion cell dysfunction in Leber hereditary optic neuropathy. Mitochondrion.

[23] Quigley HA, Dunkelberger GR, Green WR (1988). Chronic human glaucoma causing selectively greater loss of large optic nerve fibers. Ophthalmology.

[24] Weber AJ, Chen H, Hubbard WC, Kaufman PL (2000). Experimental glaucoma and cell size, density, and number in the primate lateral geniculate nucleus. Investig. Ophthalmol. Vis. Sci..

[25] Wang AY (2020). Potential mechanisms of retinal ganglion cell type-specific vulnerability in glaucoma. Clin. Exp. Optom..

[26] Swanson WH, Sun H, Lee BB, Cao D (2011). Responses of primate retinal ganglion cells to perimetric stimuli. Investig. Ophthalmol. Vis. Sci..

[27] Werner EB, Beraskow J (1979). Peripheral nasal field defects in glaucoma. Ophthalmology.

[28] Lee J, Park CK, Park HL (2021). Determinants of vessel defects in superficial and deep vascular layers in normal-tension glaucoma using optical coherence tomography angiography. Sci. Rep..

[29] Na KI, Lee WJ, Kim YK, Jeoung JW, Park KH (2017). Evaluation of optic nerve head and peripapillary choroidal vasculature using swept-source optical coherence tomography angiography. J. Glaucoma.

[30] Begg IS, Drance SM, Sweeney VP (1971). Ischaemic optic neuropathy in chronic simple glaucoma. Br. J. Ophthalmol..

[31] Kitazawa Y, Shirato S, Yamamoto T (1986). Optic disc hemorrhage in low-tension glaucoma. Ophthalmology.

[32] Park HY, Jeong HJ, Kim YH, Park CK (2015). Optic disc hemorrhage is related to various hemodynamic findings by disc angiography. PLoS ONE.

[33] Yoo E, Yoo C, Lee TE, Kim YY (2017). Comparison of retinal vessel diameter between open-angle glaucoma patients with initial parafoveal scotoma and peripheral nasal step. Am. J. Ophthalmol..

[34] Park SC (2011). Initial parafoveal versus peripheral scotomas in glaucoma: Risk factors and visual field characteristics. Ophthalmology.

[35] Enroth-Cugell C, Goldstick TK, Linsenmeier RA (1980). The contrast sensitivity of cat retinal ganglion cells at reduced oxygen tensions. J. Physiol..

[36] Drujan BD, Svaetichin G, Negishi K (1971). Retinal aerobic metabolism as reflected in S-potential behavior. Vis. Res..

[37] Suh MH (2016). Deep retinal layer microvasculature dropout detected by the optical coherence tomography angiography in glaucoma. Ophthalmology.

[38] Suh MH (2018). Deep-layer microvasculature dropout by optical coherence tomography angiography and microstructure of parapapillary atrophy. Investig. Ophthalmol. Vis. Sci..

[39] Sung MS, Heo H, Park SW (2018). Microstructure of parapapillary atrophy is associated with parapapillary microvasculature in myopic eyes. Am. J. Ophthalmol..

[40] Shin DY (2019). Association between peripapillary scleral deformation and choroidal microvascular circulation in glaucoma. Sci. Rep..

[41] Lee EJ, Kim TW, Kim JA, Kim JA (2018). Central visual field damage and parapapillary choroidal microvasculature dropout in primary open-angle glaucoma. Ophthalmology.

[42] She X (2018). Reliability of vessel density measurements in the peripapillary retina and correlation with retinal nerve fiber layer thickness in healthy subjects using optical coherence tomography angiography. Ophthalmologica.

[43] Al-Sheikh M, Tepelus TC, Nazikyan T, Sadda SR (2017). Repeatability of automated vessel density measurements using optical coherence tomography angiography. Br. J. Ophthalmol..

[44] Lee SH (2016). Reduction of the lamina cribrosa curvature after trabeculectomy in glaucoma. Investig. Ophthalmol. Vis. Sci..

[45] Quigley HA, Addicks EM, Green WR, Maumenee AE (1981). Optic nerve damage in human glaucoma. II. The site of injury and susceptibility to damage. Arch. Ophthalmol..

[46] Lee EJ, Kim TW, Kim M, Kim H (2015). Influence of lamina cribrosa thickness and depth on the rate of progressive retinal nerve fiber layer thinning. Ophthalmology.

[47] Furlanetto RL (2013). Posterior displacement of the lamina cribrosa in glaucoma: In vivo interindividual and intereye comparisons. Investig. Ophthalmol. Vis. Sci..

[48] Lee SH, Kim TW, Lee EJ, Girard MJ, Mari JM (2017). Diagnostic power of lamina cribrosa depth and curvature in glaucoma. Investig. Ophthalmol. Vis. Sci..

[49] Wu J (2021). The influence of different intraocular pressure on lamina cribrosa parameters in glaucoma and the relation clinical implication. Sci. Rep..

[50] Park HL (2021). Predicting the development of normal tension glaucoma and related risk factors in normal tension glaucoma suspects. Sci. Rep..

